# Effects of Combined Physical and Cognitive Virtual Reality-Based Training on Cognitive Impairment and Oxidative Stress in MCI Patients: A Pilot Study

**DOI:** 10.3389/fnagi.2018.00282

**Published:** 2018-10-01

**Authors:** Simona Mrakic-Sposta, Simona G. Di Santo, Flaminia Franchini, Sara Arlati, Andrea Zangiacomi, Luca Greci, Sarah Moretti, Nithiya Jesuthasan, Mauro Marzorati, Giovanna Rizzo, Marco Sacco, Alessandra Vezzoli

**Affiliations:** ^1^Consiglio Nazionale delle Ricerche (IBFM-CNR), Istituto di Bioimmagini e Fisiologia Molecolare, Milan, Italy; ^2^IRCCS Fondazione Santa Lucia, Rome, Italy; ^3^Consiglio Nazionale delle Ricerche (ITIA-CNR), Istituto di Tecnologie Industriali e Automazione, Milan, Italy; ^4^Dipartimento di Elettronica, Informazione e Bioingegneria, Politecnico di Milano, Milan, Italy; ^5^Consiglio Nazionale delle Ricerche (ITB-CNR), Istituto di Tecnologie Biomediche, Milan, Italy

**Keywords:** MCI, virtual reality, physical-cognitive training, oxidative stress, EPR

## Abstract

The growing elderly population and the increased incidence of mild cognitive impairment (MCI) and Alzheimer’s disease (AD) call for the improvement of the quality and the efficacy of the healthcare and social support services. Exercise and cognitive stimulation have been demonstrated to mitigate cognitive impairment and oxidative stress (OxS) has been recognized as a factor that contributes to the advancement of neurodegenerative diseases. Taking these aspects into account, the impact of a novel virtual reality (VR)-based program combining aerobic exercise and cognitive training has been evaluated in the pilot study proposed here. Ten patients (aged 73.3 ± 5.7 years) with MCI (Mini-Mental State Examination, MMSE: 23.0 ± 3.4) were randomly assigned to either 6 weeks physical and cognitive training (EXP) or control (CTR) group. Evaluations of cognitive profile, by a neuropsychological tests battery, and OxS, by collection of blood and urine samples, were performed before and at the end of the experimental period. The assessment of the patients’ opinions toward the intervention was investigated through questionnaires. EXP group showed a tendency towards improvements in the MMSE, in visual-constructive test and visuo-spatial tests of attention, while CTR worsened. EXP group showed a greater improvement than CTR in the executive test, memory functions and verbal fluency. No statistical significance was obtained when comparing within and between both the groups, probably due to small number of subjects examined, which amplifies the effect of the slight heterogeneity in scores recorded. Despite a greater worsening of Daily Living Activities tests, all participants reported a better performance in real life, thanks to the elicited self-perceived improvement. After training intervention OxS (i.e., reactive oxygen species (ROS) production, oxidative damage of lipids and DNA) decreased resulting in significantly (range *p* < 0.05–0.001) lower in EXP vs. CTR group. Although not conclusive, the recorded effects in the present study are promising and suggest that this proposal would be a useful tool in support of cognitive training reducing OxS too. However, further studies on larger scale samples of patients are needed.

## Introduction

In recent years, a dramatic increase of the incidence of age-related disorders due to the rise in average life-span has been recorded. One of the most common age-related pathologies is Mild Cognitive Impairment (MCI), which often represents a transition from healthy aging to Alzheimer’s disease (AD), a devastating neurodegenerative disorder so far without a cure.

MCI patients report a cognitive decline characterized by symptoms such as impaired memory, attention, orientation and executive functions, which however do not interfere remarkably with daily life activities.

Recently developed brain plasticity theories and findings about the cellular synapses reconstruction ability of nervous system due to interaction with enriched environments have spurred new researches. As a consequence, cognitive rehabilitation interventions adopting non-invasive non-pharmacological treatments have gained growing attention (Ferrucci et al., 2008; Cotelli et al., 2012; Kim et al., 2016; Marceglia et al., 2016; Chiu et al., 2017). The therapeutic goal of cognitive training is oriented to stimulate, restore or re-train the cognitive processes implied in the initiation, planning and execution of activities of daily living (ADLs), in order to reduce the side effects, maximize the patient’s ecological autonomy and quality of life. Cognitive training in MCI may stimulate pre-existing neural circuits or prompt the recruitment of alternate neural pathways prompting neuroplastic re-organization. According to a recent meta-analysis (Sherman et al., 2017) cognitive interventions adopting memory-based strategies, targeting multiple cognitive domains or using a multi-componential approach (i.e., targeting lifestyles) proved effective to improve performances on cognitive outcomes for people with MCI.

Besides, Alzheimer’s Association, based on a perspective population-based study, has established that one of the strategies to reduce cognitive decline risk and dementia development is regular physical exercise (Baumgart et al., 2015). Improvements in tests of executive function (Symbol Digit Modalities, Verbal Fluency and Stroop) were shown in a large randomized trial on aerobic exercise in MCI patients (Baker et al., 2010).

Furthermore, according to a recent systematic review (Cammisuli et al., 2017) there are evidences that aerobic exercise in MCI patients improves global cognition, logical memory, inhibitory control and divided attention. Decrease in oxidative stress (OxS) and increase in antioxidant and anti-inflammatory capacity are some of the molecular mechanisms underlining the beneficial effect of regular physical activity. Indeed, reactive oxygen species (ROS) play a dual role by means of detrimental effects linked to increased uncontrolled production, evident in the development of neurodegenerative diseases and particularly in AD (Tönnies and Trushina, 2017) vs. essential neuroprotective cellular mechanisms based on the concept of mitohormesis. The latter suggests that an exposure to intermittent sub-lethal doses of ROS generated by exercise could lead to a mitochondrial adaptation inducing mitochondrial biogenesis and an antioxidant response. The development of strategies to mitigate OxS in neurodegenerative diseases should carefully consider these aspects.

One major issue regarding usual neuro-rehabilitation programs is represented by the transferability in real life of the benefits obtained in the conventional therapeutic setting. In recent years, ICT technologies, such as virtual reality (VR), has become an increasingly used approach in neuroscience to provide an ecologically valid treatment (Coyle et al., 2015). The major features of VR are aimed at providing easy and intuitive interaction and multidimensional sensory feedback, offering the patient an opportunity to practice activities of daily living that cannot occur in conventional rehabilitation programs, through motivational activities facilitating treatment compliance and customizable on the patient’s characteristics (Rizzo and Kim, 2005; García-Betances et al., 2015b). Therefore, research on VR applied to neurorehabilitation has recently flourished, with increasing evidence of efficacy (Rizzo and Kim, 2005; Serino et al., 2017; Anderson-Hanley et al., 2018; Tieri et al., 2018).

Currently, there is limited but promising evidence on the application of VR technologies in subjects with MCI or mild dementia (Coyle et al., 2015).

In neuroscience, the computerized approach provided by VR is used in order to allow interventions in a controlled environment, able to monitor movement, cognitive and other variables (García-Betances et al., 2015a). Recently, the attention of clinicians and researchers in the field of MCI has been attracted by motor and cognitive training based on VR, which has emerged as an encouraging tool in many therapeutic and rehabilitative domains (Rizzo and Kim, 2005; García-Betances et al., 2015b). In a randomized controlled trial, Talassi et al. (2007) compared the effectiveness of a computerized training specifically developed to address different cognitive domains, with respect to physical activity in a sample of patients with MCI and mild dementia. Authors reported that significant improvements of the patients’ cognitive and affective status were obtained only in the group undertaking the computerized program, indicating that non-punctual stimulation is not able to elicit any effect.

This pilot study aims to evaluate the impact of an innovative combined physical activity and cognitive training based on VR, in MCI patients. The two main fields of investigation of this multi-domain assessment are: (1) the effects of this intervention on cognitive impairment and on OxS, assessed as indicator of the disease progression and (2) the evaluation of the user acceptability, with the final goal of verifying the potentialities of this kind of approach in counteracting MCI evolution.

## Materials and Methods

### Experimental Session

A controlled pilot study was carried out in 10 patients—4 males (M) and 6 females (F)—aged ≥65 years, with: (a) one or more test scores indicating a compromise of visuospatial abilities, (b) one or more test scores indicating mild-moderate cognitive impairment according to Mini Mental State Examination (MMSE) criteria (Folstein et al., 1975; Magni et al., 1996a,b). Exclusion criteria adopted were: cognitive and/or functional impairment affecting the ability to participate in the study; cardiovascular pathologies advising the undergoing a minimal physical training; acute pain of lower back or extremities; peripheral neuropathy; rheumatic and orthopedic diseases; inability to provide informed consent. Moreover, each subject who could potentially be enrolled had first to undergo an assessment of risk factors (cardiovascular pathologies and other co-morbidities) and an effort electrocardiogram (ECG) to exclude pathologies that may cause harm to the patients during the physical exercise. All decisions about eligibility were made before the block-randomization, and performed on the basis of cognitive decline and assessed through psychometric tests. Participants were randomized to an intervention (EXP) group (*n* = 5, 2M/3F) which underwent the intervention or to a control (CTR) group (*n* = 5, 2M/3F), who received no treatment. Three MCI patients and two patients with mild dementia (i.e., an impairment of cognition that significantly affected instrumental skills of daily life) were included in each experimental group. Individuals participating in the study provided informed written consent. This study was carried out in accordance with GCP recommendations of the Ethical Committee of IRCCS Santa Lucia Foundation, which approved the protocol (Protocol Number: CE/PROG.524, 28/09/2015). All subjects gave written informed consent in accordance with the Declaration of Helsinki.

**Figure 1:**
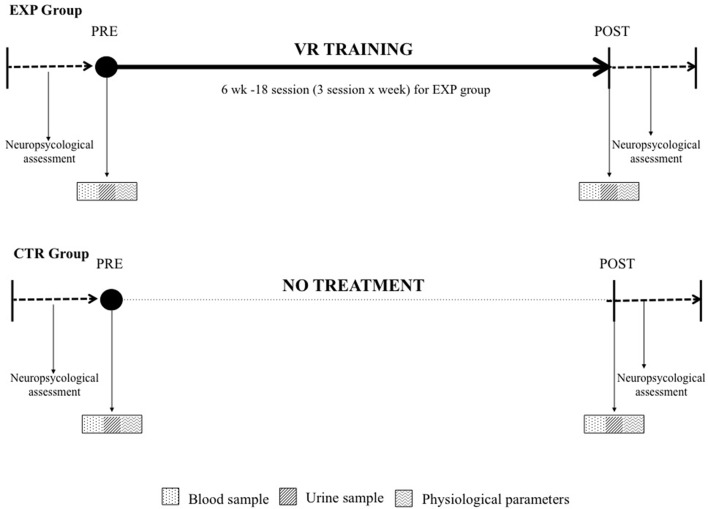
Experimental protocol of the virtual reality (VR)-based training program. The training consisted of 18 sessions for six consecutive weeks, with three sessions per week. All the assessments were carried out at baseline (PRE) and after (POST) 6 weeks for both groups (EXP—see upper part of the figure and CTR—lower part of the figure).

In order to verify the effects of the training, evaluations were performed before (PRE) and at the end (POST) of the experimental 6 weeks (see Figure 1). At these time points, anthropometric measures, cognitive profile and collection of blood and urine samples were carried out in both the experimental and the control group. Moreover, at the same time points, questionnaires to evaluate the treatment acceptability were administered to the subjects in the intervention group.

### Equipment

**Figure 2:**
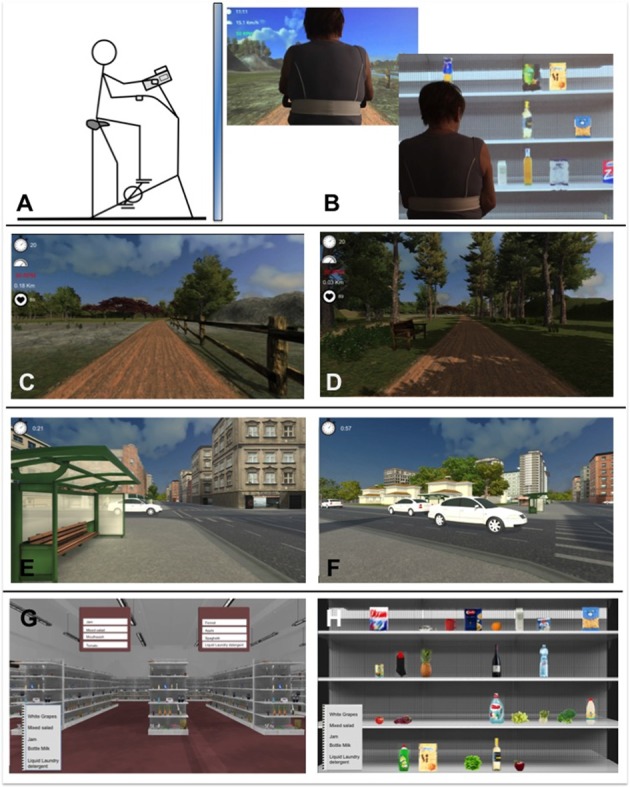
Sketch of the Physical and Cognitive Training protocol. **(A)** The cycle-ergometer. **(B)** The back view of the subject while conducting the test in virtual environments (VEs, 1,3). **(C,D)** VEs (1): “riding a bike in a park”. **(E,F)** VEs (2): “crossing roads—avoiding cars”. **(G,H)** VEs (3): “making the grocery shopping in a supermarket”.

The implemented system was designed to allow MCI patients to both perform physical exercise and train their cognitive abilities while being supported by VR means. The three virtual environments (VEs) developed enable to emulate the performance of three activities of daily living (see Figure 2). This choice has the aim of facilitating the comprehension of the tasks for patients and of enhancing the transfer of the acquired capabilities into real life. The three VEs thus represented the following scenarios: (1) riding a bike in a park, (2) crossing roads—avoiding cars and (3) grocery shopping in a supermarket. The first scenario is dedicated to the accomplishment of the physical exercise, whereas scenarios (2) and (3) are designed to provide the cognitive training. They all were developed using Unity3D. The hardware devices composing the training system are a cycle-ergometer (Cosmed EuroBike 320), a smart garment (Wearable Wellness System, Smartex), for a real time measure of heart rate (HR), a finger touch projector (EB-1430WI, Epson) and a PlayStation controller anchored on the cycle-ergometer handlebars (Sony).

VE developed in the first scenario (1) represents a trail in a park in which the user can navigate by pedalling on the cycle-ergometer. The path to follow is predefined to avoid the user from getting lost or reaching the edges of the designed scene; bends and slopes are smooth and have a large curvature to reduce the occurrence of sickness as much as possible. To increase the user’s presence in the VE, the realism of the scene requires that the visual flow is synchronized with the cycling velocity in real-time, when the wind is blowing the tree leaves and grass move, wild animals appear on the trail sides or in the sky. Realistic 3D sounds reproducing birds’ chirp and the wind have been are added to the scene too. Indicators showing the bike velocity and revolutions-per-minute, the user’s HR (measured thanks to the smart garment) and the elapsed time are shown as on-screen text to provide the patients with general information about the exercise and to help them to maintain the right cycling speed (see Training Protocol).

The second virtual environment (2) represents an urban scenario that the patient is supposed to reach after the physical activity performed in the park. In this scenario, the user—on the cycle-ergometer—has to cross several traffic-congested crosses by stopping on the edge of the sidewalk and checking if the route is safe from moving cars. Cars are generated randomly and move at different velocities. 3D sounds simulating an accident are reproduced if a car hits the user while he/she is crossing. After the accident, the user is brought in a safe-position and can restart to pedal after a few seconds, when the cars disappear from the cross. To brake and to turn the point of view, the patient has to use the PlayStation controller: respectively, the X button and the right joystick; to move toward the other side, he/she has to restart cycling.

The third virtual environment (3) represents a supermarket. Before entering, the user is shown a list of five grocery items that he/she has to buy by touching the projected screen to make his/her selection. The purchase of each item is performed by completing two tasks: (3.1) selecting the right aisle and then (3.2) picking the right product from the shelves. Five different levels of difficulty were implemented for both tasks. The increase in difficulty is obtained mainly by the increase of distractors and of their similarity and by their position on the shelf. For the aisle task, a further stimulus to promote language and attentional training is obtained by the introduction of a word that is orthographically or semantically similar to the target object name (from Level #3). For the shelf task, higher levels are characterized by the presence of different formats (Level #4) and discounted versions of the same product (Level #5), so that the attentional and visuospatial demands are increased.

The system intervenes if the user commits an error or does not interact with the VE for more than 20 s (latency) and gives him/her a hint to help in proceeding to the next task.

### Training Protocol

The intervention group performed the physical and cognitive training for 6 weeks, 3 sessions/week (18 sessions total). The structure of VR-based training session, carried out in a quiet room, was the same for each participant and lasted approximately 40–45 min long: 15 (for the first 3 weeks) or 20 min of cycling in the virtual park, about 5 min to perform five crossroads passing and 20 min of shopping at the supermarket. Before the beginning of the training program, the participants were assisted in a preliminary session so that they could familiarize with VR technologies and learn how to interact within the three virtual environments.

Cycling exercise intensity was set as to correspond to about 65%–70% of individual maximal HR, calculated on the basis of the age-predicted value. During the training session, the patients were instructed to keep the cycling velocity between 60 and 70 revolutions per minute; the cycle-ergometer workload was continuously adjusted (thanks to a digital controller based on a proportional-integral feedback mechanism) with the aim of maintaining the target HR at a constant frequency.

Dealing with the supermarket scenario (3), after having completed three subsequent shopping sessions making one error at the most (or waiting once for more than 20 s), the subject could move to the next level of difficulty. When patients completed Level #5 for three times without committing errors or latencies, the training was restarted from the first level in which until he/she failed for the first time with the list displayed on the screen. During this second cycle, the shopping list was kept hidden with the aim of training patients’memory too.

### Assessment of Cognitive Response

An in-depth cognitive assessment was performed by a trained neuropsychologist at PRE and POST intervention. Each subject was assessed by the same evaluator at PRE and POST, in order to avoid inter-rater variability effects.

The entire neuropsychological battery was composed by an initial screening test for cognitive abilities (MMSE, Magni et al., 1996a,b) and a subsequent extensive battery of specific scales for each cognitive domain.

Episodic verbal memory was evaluated by Immediate Recall and Delayed of Rey Auditory (RAVLT_I and RAVLT_D, Carlesimo et al., 1996): in the RAVLT_I, a list of 15 words is presented to the subject, which he/she has to memorize and repeat. The task is repeated five times and the number of words correctly recalled is recorded. The RAVLT_D requires the recall, after 15 min, of the same words.

Visuo-spatial functions were assessed with the Rey-Osterrieth Complex Figure Test (ROCFT; Caffarra et al., 2002), the Attentional Matrices Test (AM; Spinnler and Tognoni, 1987), and the Trail Making Test A (TMT-A, Giovagnoli et al., 1996). The ROCFT is commonly used for the evaluation of constructive apraxia, and requires to copy a geometric drawing composed of 18 sub-elements: the correct execution and collocation of the sub-elements are both criteria for scoring. AM inquires selective visuo-spatial attention and requires to delete, as quickly as possible, all the numbers identical to the targets among distractors. In the TMT-A, the subject must perform a visuo-spatial search to connect in ascending order, with a stroke of pen, a series of 25 numbers presented in a random distribution on a sheet.

The executive functions were investigated with the Frontal Assessment Battery (FAB, Appollonio et al., 2005) which evaluates categorization, planning, inhibition, sensitivity to interference and environmental autonomy and with the Trail Making Test B (TMT-B, Giovagnoli et al., 1996) which requires to combine alternatively numerical and alphabetical stimuli, in order to explore visual-motor coordination, set-shifting and mental flexibility.

Lexical finding skills were assessed with the Verbal Fluency test (VF, Novelli et al., 1986), in which the subject must name the greatest possible number of words belonging to a given semantic category in a minute.

Finally, the subject’s independency in daily life activities was inquired with the caregiver, using the Functional Activity Questionnaire (FAQ).

### Assessment of Oxidative Stress

#### ROS Determination

ROS production rate was determined adopting a recently developed Electron Paramagnetic Resonance (EPR) mini-invasive method (Mrakic-Sposta et al., 2012, 2014, 2015, 2017), using 50 μL of capillary blood taken from the fingertip and immediately treated with CMH (1-hydroxy-3-methoxycarbonyl-2,2,5,5-tetramethylpyrrolidine) probe solution (1:1). For data acquisition 50 μL of the obtained solution was put in a glass EPR capillary tube (Noxygen Science Transfer & Diagnostics, Germany) placed inside the cavity of a X-band EPR instrument (E-Scan-Bruker BioSpin, Billerica, MA, USA). Acquisition parameters were determined previously (Mrakic-Sposta et al., 2012). A Temperature and Gas Controller “Bio III” unit, interfaced to the spectrometer, was used in order to stabilize sample temperature at 37°C. Absolute ROS production rate (μmol.min^−1^) was obtained converting relative quantitative determination, allowed by EPR measurements, by adopting the stable radical CP• (3-Carboxy2,2,5,5-tetramethyl-1-pyrrolidinyloxy) as external reference. The software supplied by Bruker (Win EPR System, V. 2.11) was adopted for acquisition parameters and spectra handling.

#### Antioxidant Capacity

A capillary blood sample (10 μL) was used in order to assess Blood Reducing Capacity by mean of a commercial EDEL potentiostat electrochemical analyzer (Edel Therapeutics, Switzerland) equipped with a redox sensor in a three-electrode arrangement able to respond to all water-soluble compounds in biological fluids, which can be oxidized within a defined potential range (Liu et al., 2005, 2006). The blood sample was loaded onto a chip and the result was then pseudo-titrated to account for the most biologically relevant antioxidants (Tacchini et al., 2013). Data were expressed in nW.

#### Enzymatic Assays

Urine samples were collected in a sterile container and stored in multiple aliquots at −80° C until assessment performed within 2 weeks from collection.

8-hydroxy-2-deoxy Guanosine (8-OH-dG), as a marker of oxidative DNA damage, and 8-isoprostane (8-iso), an established marker of lipid peroxidation, were assessed by commercially enzyme immunoassay kit (Cayman Chemical, Ann Arbor, MI, USA). The procedures described in detail by the manufacturer were followed.

As the collection of the 24 h urine was not possible, urinary parameters were standardized based on the amount of the excreted creatinine, in order to avoid the well-known considerable changes occurring over time. Indeed, the excretion rate of creatinine keeps relatively constant in the absence of renal disease. Creatinine concentration was assessed by commercial enzymatic assay kit (Cayman Chemical, Ann Arbor, MI, USA). The procedures described in detail by the manufacturer were performed.

### Assessment of the Treatment Acceptability

Acceptability is defined as the degree to which non-professional stakeholders found an intervention to be fair, reasonable, intrusive and consistent with treatment expectations (Wolf, 1978; Kazdin, 1980). It refers to the overall evaluation of the applied procedures and constitutes an important dimension of the treatment evaluation, besides treatment efficacy and effectiveness (Kazdin, 1980). In this study, the assessment of the patients’ opinions toward the intervention was made through a semi-structured interview administered at the beginning of the training program and at the end of week 6 by a psychologist and a biomedical engineer. In particular, closed questions were used to enable the collection of precise information about a specific topic (i.e., “was the smart garment comfortable?”), whereas the open ones had the goal of better exploring patients’ subjective perceptions and feelings. Indeed, open questions constituted as a “topic guide” to ensure the completeness and comparison between different responses, but a high level of flexibility was kept to indulge each participant’s attitude. Before starting the questionnaire administration, the purpose of the interview and the assurance that all opinions were valuable and confidential were disclosed to the patients to encourage honest feedback.

Answers were transcribed by one of the administrators during the interview. Descriptive statistics (median and interquartile ranges) were used to provide a summary of the data gathered through closed questions. Thematic analysis was subsequently performed to further investigate patients’ subjective perceptions, gathered through open questions and free comments.

### Statistical Analysis

For the purpose of comparability between different neuropsychological tools, the scores of all the administered tests were standardized according to their normative mean scores and standard deviations (Novelli et al., 1986; Spinnler and Tognoni, 1987; Carlesimo et al., 1995; Giovagnoli et al., 1996; Magni et al., 1996a,b; Caffarra et al., 2002; Appollonio et al., 2005). Only three participants, in PRE, were able to complete the TMT-B, so, this scale was excluded from subsequent comparisons.

GraphPad Prism package (GraphPad Prism 7, GraphPad Software Inc., San Diego, CA, USA) was used for statistical analysis. All statistical analyses were performed using non-parametric tests, due to the small sample size. The effect of the combined physical and cognitive VR-based training on the neuropsychological assessments and the OxS levels between the CTR and the EXP groups were compared using Mann Whitney U-test for independent samples. In detail, such test was used to compare the scores at PRE and POST conditions for OxS, and the change in the scale scores from PRE to POST conditions for psychometric tests, between EXP and CTR groups.

Non-parametric Wilcoxon matched pair signed rank test was adopted to compare the change in the scale scores from PRE to POST conditions within the EXP and CTR groups. For cognitive and functional scores ITT-LOCF analyses are presented. Data are presented as mean ± SD and a *p* < 0.05 was considered statistically significant.

## Results

The socio-demographic and clinical characteristics of the sample are substantially comparable to those used to assess the effectiveness of interventions aimed at slowing down cognitive decline (mean age = 73.3 ± 5.6 years, mean schooling = 7.6 ± 4.4 years, mean MMSE = 23.0 ± 3.4).

A drop out of one patient both in EXP and CTR group was recorded for reasons independent from the intervention. Adherence, for the remaining patients in the EXP group, was judged good (more than 75%, reaching 89% in two cases) for three of them, and partial (more than 50% and less than 75%) for one patient (67%).

The age, the anthropometric characteristics and the physiological parameters (i.e., peripheral oxygen saturation (SpO_2_), HR) of the subjects, separated in the examined groups, are reported in Table 1. No significant differences between the two groups examined at baseline were found.

**Table 1:** Baseline characteristics.

	EXP	CTR
**Anthropometric Characteristics**		
Age (years)	72.00 ± 5.15	74.60 ± 6.43
Height (m)	1.63 ± 0.11	1.64 ± 0.59
Weight (kg)	62.63 ± 7.69	57.82 ± 7.54
BMI	23.64 ± 2.02	21.28 ± 1.89
Fat Mass (kg)	15.82 ± 4.77	12.65 ± 1.79
Free Fat Mass (kg)	47.10 ± 9.66	45.76 ± 8.08
Total Body Water (kg)	36.78 ± 8.02	33.5 ± 5.90
**Physiological Parameters**		
SaO_2_ (%)	97.00 ± 0.71	97.20 ± 0.84
HR (bit/min)	78.40 ± 11.46	71.80 ± 7.56

*Mean (±SD) values of the anthropometric features and physiological parameters measured before the intervention in experimental (EXP) and control (CTR) group. BMI, Body Mass Index; SaO_2_, arterial Oxygen Saturation; HR, Heart Rate*.

### Assessment of Cognitive Response

The sample was composed of subjects with a mild deterioration of global cognition that is classifiable as mild (mean MMSE score = 23.0 ± 3.4).

The subjects globally presented a slight impairment in all the investigated cognitive domains (Table 2) and a moderate impairment in the visuo-constructive and executive functions (ROCFT standardized scores were −2.15 ± 2.85 in EXP and −2.71 ± 2.45 in CTR; FAB standardized scores were −1.66 ± 1.49 in EXP and −2.29 ± 2.59 in CTR). A mild functional impairment was observed in both groups (FAQ scores 8.80 ± 7.19 for EXP and 10.60 ± 8.08 for CTR). No statistically significant differences were observed between the scores obtained by the EXP and CTR group in any of the scales administered at PRE.

**Table 2:** Pre-post assessment.

	PRE			Delta score (POST—PRE)		
	EXP	CTR	*p*	EXP	CTR	*p*
MMSE	−1.63 ± 1.52	−1.68 ± 1.46	*0.690*	0.17 ± 1.58	−0.17 ± 0.48	*0.690*
RAVLT_I	−1.18 ± 1.08	−0.68 ± 1.37	*0.421*	0.68 ± 1.40	0.06 ± 0.25	*0.548*
RAVLT_D	−1.11 ± 1.22	−1.19 ± 1.37	*1.000*	0.63 ± 0.63	0.06 ± 0.35	*0.095*
ROCFT	−2.15 ± 2.85	−2.71 ± 2.45	*0.690*	0.17 ± 1.74	−0.07 ± 0.23	*0.690*
AM	−0.35 ± 0.6	−1.75 ± 2.25	*0.421*	0.06 ± 0.69	−0.14 ± 0.99	*0.690*
TMT-A	−0.14 ± 0.67	−1.87 ± 3.42	*0.310*	−0.03 ± 0.57	0.00 ± 0.59	*1.000*
FAB	−1.66 ± 1.49	−2.29 ± 2.59	*1.000*	1.00 ± 0.82	0.54 ± 1.31	*0.310*
VF	−0.14 ± 0.83	−1.06 ± 1.28	*0.095*	0.28 ± 0.97	0.11 ± 0.44	*1.000*

*Mean standardized scores at the baseline (PRE) for the experimental (EXP) and control (CTR) groups and mean change in standardized scores between the baseline and the end of experimental phase calculated as delta scores (POST—PRE; except for Trail Making Test A that implies decreasing scores in relation to better performance, for which the changes were calculated as PRE—POST). MMSE, Mini-Mental State Examination; RAVLT_I, Immediate Recall of Rey Auditory Verbal Learning Test; RAVLT_D, Delayed Recall of Rey Auditory Verbal Learning Test; ROCFT, Rey-Osterrieth Complex Figure Test; AM, Attentional Matrices; TMT-A, Trail Making Test A; FAB, Frontal Assessment Battery; VF, Verbal Fluency test*.

At POST, EXP showed a slight improvement in MMSE, ROCFT, FAB and AM, while CTR worsened. Both groups showed improvement over time in memory tests and word finding for EXP the improvement was higher, but not statistically different from that of CTR. In the TMT-A, EXP and CTR resulted substantially stable in time (Table 2).

Both groups showed a deterioration of ADLs between PRE and POST. For the EXP this deterioration was more pronounced (1.80 ± 4.60 at the FAQ) than that of the CTR (1.00 ± 1.73). However, this variation has been heavily influenced by the performance of a single subject belonging to the EXP, who lost 10 FAQ-points due to a marked deterioration of global health. The same subject was re-evaluated within the Cognitive Disorders Center 3 months after POST and showed an improvement in the FAQ scores of 6 points, compared to the PRE. Excluding that subject as an outlier, EXP obtained a slight improvement of 0.25 ± 0.5 in FAQ scores.

None of the comparisons within and between groups reached statistical significance at the Wilcoxon Matched Pair Test and at the Mann-Whitney U-Test, reasonably due to small sample size, which amplifies the effect of the slight heterogeneity in scores between subjects, even if a weak effect of the treatment on global cognition, on visuo-constructive abilities, on visuo-spatial attention and, in particular, on executive functions was observed.

### Assessment of Oxidative Stress

Comparing every baseline value of the examined biological parameters, no significant difference between EXP and CTR group was recorded.

**Figure 3:**
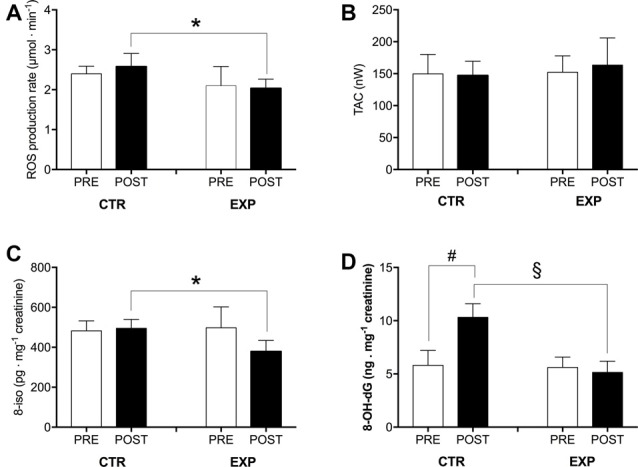
The influence of physical and cognitive training on oxidative stress (OxS) is shown by the histogram plots of: **(A)** reactive oxygen species (ROS) production rate (μmol · min^−1^), **(B)** total antioxidant capacity (TAC; nW), **(C)** lipids peroxidation (8-iso; pg.mg^−1^ creatinine) and **(D)** DNA damage (8-OH-dG; ng.mg^−1^ creatinine) obtained from capillary blood and urine samples in the control (CTR) and experimental (EXP) groups at PRE and POST (after 6 weeks). Results are expressed as mean ± SD. Statistically significant differences symbols: *p < 0.05, ^#^*p* < 0.01, and ^§^*p* < 0.001.

As previously reported (Marzorati et al., 2016), an increase (+8%) in ROS production was recorded at POST in the CTR group; on the contrary in the EXP group a decrease (−4%) was observed. Comparing the two experimental groups at POST, ROS production rate resulted statistically (*p* < 0.05) lower in EXP vs. CTR (2.03 ± 0.23 vs. 2.58 ± 0.33 μmol · min^−1^, respectively Figure 3A).

A similar trend was observed in 8-iso concentration: compared to CTR, in the EXP group after training intervention 8-iso resulted statistically (*p* < 0.05) lower (495.20 ± 44.56 vs. 379.30 ± 54.82 pg·mg^−1^ creatinine, respectively; Figure 3C).

8-OH-dG concentration significantly increased (*p* < 0.01) respect to basal value in the CTR group (5.80 ± 1.40 vs. 10.32 ± 1.28 ng·mg^−1^ creatinine) and was significantly (*p* < 0.001) lower in the EXP group with respect to the CTR after training intervention (5.14 ± 1.05 vs. 10.32 ± 1.28 ng·mg^−1^ creatinine, respectively; Figure 3D).

On the contrary after 6 weeks of training, no differences were found comparing the relative post values in the CTR and EXP groups in the TAC level (CTR: 149.75 ± 30.44 vs. post: 147.25 ± 22.20 nW; EXP: 152.25 ± 25.55 vs. post: 163.50 ± 42.35 nW; Figure 3B).

### Assessment of Treatment Acceptability

Results of the closed questions are presented in Table 3 according to the Likert scale proposed, with range from 0 (not at all) to 4 (very much).

**Table 3:** Results (median and iqr) of the questionnaire administered after the sixth week of training.

		Score
General Satisfaction	Are you satisfied with this training?	2.0 (2.0–2.5)
	Did this training meet your expectations?	2.5 (2.0–3.0)
Park/Road-crossing scenarios	Did you enjoy pedaling?	2.5 (2.0–3.0)
	Did you get tired?	1.0 (0.0–1.0)
	Was it difficult to keep the required velocity?	0.0 (0.0–0.0)
	Did you feel comfortable wearing the smart garment?	4.0 (4.0–4.0)
	Was it comfortable to use the joystick?	3.0 (1.8–4.0)
	Did you enjoy looking at the park while pedaling?	3.0 (2.8–3.0)
	Did you get sick?	0.5 (0.0–0.5)
	Did you get bored?	0.0 (0.0–0.0)
	Did you get anxious?	0.0 (0.0–0.0)
Supermarket Scenario	Did you enjoy doing the shopping?	2.3 (1.8–3.0)
	Was it comfortable to interact with the touch screen?	2.0 (2.0–3.0)
	Was the shopping list clear?	3.0 (2.8–3.5)
	Was the guiding voice clear?	3.0 (2.9–3.5)
	Were the hints useful?	3.0 (3.0–3.0)
	Did you encounter specific complications?	1.5 (1.0–1.5)
	Did you get sick?	0.0 (0.0–0.0)
	Did you get bored?	0.0 (0.0–0.0)
	Did you get anxious?	0.0 (0.0–0.0)

*The evaluation was based on a Likert scale whose elements indicated: not at all (0), not really (1), neutral (2), somewhat (3) and very much (4). Results in this table were previously published in Arlati et al. (2017)*.

As highlighted in the table, patients expressed in general a neutral position towards the training and a positive confirmation of their expectations consisting, according to open questions, in an improvement or, at least, to maintain their current status (2,5). These data showed that in general, the intervention was judged acceptable and enjoyable by all the subjects. The interaction with the technology was easy and intuitive for all of them and only few side effects were reported. The setup was defined comfortable and changing clothes to wear the smart garment was not an issue. In particular, considering the scenarios supporting the physical training (park and road-crossing), participants scored positively with the joystick use and did not complain about the difficulty of the physical exercise, also in terms of maintaining the speed required (0). Also in the supermarket scenario for the cognitive training, the different visual and auditory supports (shopping list, guiding voice and hints) were judged clear and useful (3).

Difficulties related to the cognitive tasks were mainly related to graphical issues or products’ design, which did not match the packages the participants used to buy. Moreover, answers from participants reported that the different scenarios did not elicit negative feelings as anxiety and boredom (0) except for a slight sense of sickness in the park and road crossing scenarios (0.5). Some interesting considerations on acceptability arose also from the open questions analysis, confirming and deepening specific aspects highlighted in the Table 3. First, all participants stated that they would continue with the training, if possible, and most of them also at their own home with a proper setup, thus confirming a positive acceptance level. Concerning expectations, all of them reported to have received benefits from the intervention, both in terms of motivation and perceived improvements. It is important to underline that, according to the self-perceived improvements, subjects of EXP group reported better performance in real life regardless of real improvements from results of cognitive tests, probably due to a related reduced level of anxiety.

## Discussion

Dementia pathogenesis is a multifactorial disorder where mechanisms involved are not fully understood, but an important role appears to be played by the interaction of genetic and environmental factors.

The disease prevention appears to be possible, as many factors involved in the onset and progression of dementia are modifiable or susceptible to management. Therefore, encouraging the implementation of preventive measures throughout life may be most a effective strategy. Indeed, in Europe, three major multi-center trials for the prevention of cognitive decline and dementia have been launched, and they all foresee multicomponent interventions (Vellas et al., 2014; Ngandu et al., 2015; Moll van Charante et al., 2016).

The evidence suggests that a multi-dimensional approach aimed at increasing physical activity levels and stimulating cognitive functions may have an effect on slowing cognitive decline in a population of elderly people at risk of dementia.

The novelty of this VR-based system supporting physical and cognitive training enabled to conduct a preliminary evaluation of the efficacy and of the acceptability of an innovative non-pharmacological system able to mitigate some of the dementia effects, by means of exploiting VR technologies for the training of both functions in elderly with MCI.

The combination of training and VR technologies, adopted in the presented protocol, was revealed able to achieve the expected goals, promoting a tendency towards improvement in people affected by cognitive impairments. The application allowed to train important cognitive functions such as executive function, navigation, planning and memory and support procedures for reducing behavioral and psychological symptoms of MCI and early-stage AD patients. Obtained results are in agreement with recent reviews (Coyle et al., 2015; Hill et al., 2016) on computerized and VR-based training of cognitive abilities in MCI population, which showed that attention, executive functions and memory are the domains that can be better addressed with this type of training.

Even if this study has average sample and time of intervention smaller than that of others (Etnier et al., 2006) evaluating the effects of physical exercise on cognition, the presented results suggest that the adopted training protocol was actually able to affect MMSE tasks and to increase the global cognition levels of MCI. Anyways, according to the above-mentioned review, a longer intervention would be more suitable to improve cognition in older subjects incrementing physical activity level.

Numerous evidences support the role of OxS in the development of AD. Indeed, lipid and DNA damage caused by OxS is closely associated with the development of AD (Nunomura et al., 2006) and very strong correlations among lipid peroxidation, antioxidant enzymes and senile plaques in AD brains are reported (Feng and Wang, 2012).

OxS is characterized by an imbalance between ROS production and antioxidant defense system which are responsible for the removal of ROS (Harman, 1981), both systems are considered to have relevant roles in neurodegeneration and cognitive decline, processes age-related. A decrease in plasma antioxidant defense mechanism was reported that it was associated with memory impairments related to aging (Haider et al., 2014). Thus, the development of strategies to mitigate OxS in neurodegenerative diseases is relevant. The reduction of an uncontrolled ROS production mitigating the deleterious effects should be positively considered.

Although not conclusive, the recorded effects in the present study (i.e., reduction of ROS production and oxidative damage of lipids and DNA), support the evidence that the adoption of physical and cognitive training decreases OxS levels, and speculatively, this would be associated with the slight improvement in some cognitive functions.

Concerning user acceptability, qualitative data retrieved from the questionnaires and the subjects’ comments revealed high levels of engagement and motivation, enabled mainly by the use of the VR technologies that they defined as a new way to approach the cognitive training. Feedbacks on the VEs design revealed appreciation, leading to the conclusion that the training was well accepted by all the patients, who—in the majority of the cases—would continue with the program also at home. Having reached high levels of acceptability of the proposed technology-based treatment is indeed a promising result. In fact, acceptability represents a key issue for promoting the successful employment of innovative technologies in therapy, especially when dealing with older population (Dillon and Morris, 1996).

Finally, it is important to highlight that the subjects enrolled in the experimental group reported to “feel better” and to have reduced their levels of anxiety during the accomplishment of common ADLs. This feeling, though self-perceived and not supported by psychometric results, probably due to the small sample, enabled a real improvement of their quality of life (QoL). Subjective perceptions have been recognized as one of the outcomes of the rehabilitation path, and several studies have shown how this aspect can contribute to increase patients’ satisfaction (Cicerone et al., 2000; Dohnke et al., 2005; Lexell et al., 2014) and self-efficacy, leading indeed towards more autonomy and more effectiveness in the performance of several behaviors (Grembowski et al., 1993).

## Limitations

Limitations of this clinical trial are linked to its pilot study characteristics (i.e., small sample and a short period of treatment). Moreover, the multimodal approach adopted, including treatment with multiple compounds with diverse properties that could improve several mechanisms and functions, was not compared with another active intervention (e.g., a matched cycle-ergometer training program) but without the VR component, therefore we cannot estimate neither the impact of every single component nor the additive effects. Long-term follow-ups are indispensable to evaluate the rehabilitation efficacy too. In spite of the small number of patients the strength of this study includes the supervised exercise program, a high rate of adherence to the intervention and the use of validated outcome measures. Lack of monitoring of the physical activity outside the experimental sessions and the lack of a social activity program in the control group weaken the study too.

## Conclusion

The potentialities of the proposed innovative approach have been demonstrated, even if this study should be considered as a proof-of-concept that requires further developments.

Results obtained concerning both the efficacy and the acceptability of the novel VR-based combined training in MCI populations are encouraging, but a greater number of participants are required to support the preliminary data with statistical significance. Other future challenges foresee the inclusion of a follow-up phase to provide more consistent results and highlight the long-term effectiveness of the proposed combined program, which could represent a solution to the development of a future home-based training service.

## Author Contributions

SM-S planned and conducted the experiment, analyzed data and drafted the manuscript and figures. SD and FF administered the cognitive tests, conducted the experiment, analyzed data and reviewed the manuscript. SA, AZ, LG and SM designed and developed the virtual environments and reviewed the manuscript. SA and AZ assessed the treatment acceptability. SM and NJ conducted the experiment and reviewed the manuscript. MM designed training protocol, reviewed the manuscript. GR reviewed the manuscript. AV coordinated research protocol, conducted the experiment and finalized the manuscript.

## Conflict of Interest Statement

The authors declare that the research was conducted in the absence of any commercial or financial relationships that could be construed as a potential conflict of interest.
